# Induced pluripotent stem cells can improve thrombolytic effect of low-dose rt-PA after acute carotid thrombosis in rat

**DOI:** 10.1186/s13287-021-02615-z

**Published:** 2021-10-21

**Authors:** Hsi-Lung Hsieh, Ching-Chung Liang, Cheng-You Lu, Jen-Tsung Yang, Chiu-Yen Chung, Yu-Shien Ko, Tsong-Hai Lee

**Affiliations:** 1 Department of Nursing, Division of Basic Medical Sciences, Research Center for Chinese Herbal Medicine, and Graduate Institute of Health Industry Technology, Chang Gung University of Science and Technology, Taoyuan, Taiwan; 2 Female Urology Section, Department of Obstetrics and Gynecology, Chang Gung Memorial Hospital, Linkou Medical Center, and College of Medicine, Chang Gung University, Taoyuan, Taiwan; 3 Cardiovascular and Mitochondrial Related Disease Research Center, Hualien Tzu Chi Hospital, Buddhist Tzu Chi Medical Foundation, Hualien, Taiwan; 4 Department of Neurosurgery, Chia-Yi Chang Gung Memorial Hospital, Chia-Yi, Taiwan, and College of Medicine, Chang Gung University, Taoyuan, Taiwan; 5 The First Cardiovascular Division, Department of Internal Medicine, Chang Gung Memorial Hospital, Linkou Medical Center, and College of Medicine, Chang Gung University, Taoyuan, Taiwan; 6 Stroke Center and Department of Neurology, Chang Gung Memorial Hospital, Linkou Medical Center, and College of Medicine, Chang Gung University, No. 5, Fu-Hsing Street, Kweishan, Taoyuan, 333 Taiwan

**Keywords:** Carotid thrombosis, Endothelium, Stem cell, Thrombolysis, Tissue plasminogen activator

## Abstract

**Background:**

Intravenous thrombolysis using recombinant tissue plasminogen activator (rt-PA) is the standard treatment for acute ischemic stroke. Standard-dose rt-PA (0.9 mg/kg) is known to achieve good recanalization but carries a high bleeding risk. Lower dose of rt-PA has less bleeding risk but carries a high re-occlusion rate. We investigate if induced pluripotent stem cells (iPSCs) can improve the thrombolytic effect of low-dose rt-PA (0.45 mg/kg).

**Methods:**

Single irradiation with 6 mW/cm^2^ light-emitting diode (LED) for 4 h at rat common carotid artery was used as thrombosis model according to our previous report. Endothelin-1 (ET-1), intercellular adhesion molecule-1 (ICAM-1), and interleukin 1 beta (IL-1 beta) were used as the inflammatory markers for artery endothelial injury. Angiopoietin-2 (AP-2), brain-derived neurotrophic factor (BDNF) and vascular endothelial growth factor (VEGF) were examined in artery wall and iPSCs culture. Animal ultrasound was used to evaluate the stenosis degree of common carotid artery before and at 2 h, 24 h, 4 days and 7 days after LED irradiation.

**Results:**

After LED irradiation alone, there was a persistent occlusion from 2 h to 7 days. Standard-dose rt-PA alone could recanalize the occluded artery from 24 h to 7 days to stenotic degree ≤ 50%. Low-dose rt-PA or 1 × 10^6^ mouse iPSCs alone could not recanalize the occluded arteries from 2 h to 7 days. Combination use of low-dose rt-PA plus 1 × 10^6^ mouse iPSCs caused better recanalization from 24 h to 7 days. ET-1, ICAM-1 and IL-1 beta were strongly expressed after LED irradiation but reduced after iPSCs treatment. AP-2, BDNF and VEGF were rarely induced after LED irradiation but strongly expressed after iPSCs treatment. In vitro study showed iPSCs could express AP-2, BDNF and VEGF.

**Conclusion:**

The adjuvant use of iPSCs may help improving the thrombolytic effect of low-dose rt-PA by suppressing inflammatory factors and inducing angiogenic trophic factors. Stem cells could be a potential regimen in acute thrombolytic therapy to improve recanalization and reduce complications.

**Supplementary Information:**

The online version contains supplementary material available at 10.1186/s13287-021-02615-z.

## Introduction

Stroke is the second leading cause of death and carries a severe socioeconomic burden worldwide [1]. The management of stroke depends on prevention to decrease the occurrence and early treatment to improve the outcome of stroke. In acute ischemic stroke treatment, intravenous recombinant tissue plasminogen activator (rt-PA) is used within 3–4.5 h [2, 3], and the use of intra-arterial mechanical thrombectomy can be prolonged to within 6–24 h [4, 5]. The rt-PA treatment has narrow therapeutic time window and carries a high bleeding risk with 10 folds higher than no rt-PA treatment [3].

The clinical trial of ENCHANTED suggested low-dose rt-PA (0.6 mg/kg) could be noninferior to standard-dose rt-PA (0.9 mg/kg) regarding death and disability at 90 days with less symptomatic intracerebral hemorrhages in patients with acute ischemic stroke [6]. A recent meta-analysis also found the effectiveness of low-dose rt-PA (0.6–0.9 mg/kg) is comparable to standard-dose rt-PA (0.9 mg/kg) with improved neurologic function and reduced mortality and intracerebral hemorrhage [7]. Previous studies have investigated the effect of adjuvant medications to improve rt-PA treatment in acute ischemic stroke. However, the addition of low-molecular weight heparin [8] was found to cause increased bleeding risk, and the addition of aspirin resulted in early deterioration [9]. Although rt-PA can induce artery recanalization, the re-occlusion rate was reported around 20–34%, which may be due to endothelial injury with platelet activation [10].

Induced pluripotent stem cells (iPSCs) can be generated from mouse embryonic and adult fibroblast cultures with defined factors [11] and can serve as a potential of stem-cell therapy for numbers of disease [12]. The iPSCs therapy is found to provide trophic factors, increase angiogenesis and neurogenesis, and provide new cells for tissue repair in the treatment against ischemic stroke [13]. Intracerebral transplantation of human iPSCs can ameliorate behavioral and pathological deficits [14] and reverse behavioral deficits in experimental stroke models [15, 16]. These studies suggest that iPSCs may be beneficial for the recovery of brain tissue injury.

Intracoronary infusion of stem cell combined with nanoburning has been suggested as a potential technique to improve advanced atherosclerosis by plaque destruction and vessel wall restoration [17]. In sheep carotid artery, the coating of a synthetic vascular graft with fibronectin and stem cell homing factor to stimulate the attraction of circulating stem cells can improve endothelialization and reduce intimal hyperplasia and thrombosis [18]. Our previous study using low-dose rt-PA (0.45 mg/kg) therapy in combination with focused ultrasound and a neuroprotective agent, BNG-1, showed an improved thrombolytic effect comparable to standard-dose rt-PA (0.9 mg/kg) in a rat model of acute carotid thrombotic occlusion [19]. However, the use of focused ultrasound may cause local injury to soft tissue with scarring around carotid artery.

In considering the benefit of stem cell treatment on chronic atherosclerosis, the present study wishes to investigate if iPSCs may have effect in acute thrombotic stroke by acting as an adjuvant to low-dose rt-PA (0.45 mg/kg) to improve recanalization and reduce the risk of re-occlusion using our rat model of acute carotid thrombotic occlusion [19].

## Methods

### Animals

Male Sprague–Dawley (SD) rats weighing 300–350 g and aged 12–14 weeks were purchased from BioLASCO Taiwan Co., Ltd (http://www.biolasco.com.tw/index.php/tw/) and bred in the animal center of our institute with 2 animals in one laboratory cage. All animals were given tap water ad libitum and were maintained in a temperature and humidity-controlled room on a 12-h light/dark cycle. The animal experiments were approved by our Institutional Animal Care and Use Committee (No.: IACUC 2016121923). All procedures were performed in accordance with the National Institute of Health Guide for the Care and Use of Laboratory Animals (NIH Publications No. 80-23) revised in 1996 and in compliance with the ARRIVE guidelines (Animal Research: Reporting in Vivo Experiments) for how to REPORT animal experiments. Efforts were made to minimize suffering including giving postoperative analgesia (0.3–0.5 mg/100 g Meperidine) when there was a decrease of appetite or arching of back due to pain. Surgical wound was treated with gentamycin ointment to prevent infection. Efforts were also made to reduce the number of animals by sample size calculation and utilize alternatives to in vivo techniques, if available.

### Acute carotid thrombotic occlusion model

According to our previous study [20], acute thrombotic occlusion was induced in rat common carotid artery (CCA). The rats were carefully handled to calm down the stress and quickly anesthetized with 3% isoflurane in a mixture of 30% oxygen and 70% nitrogen, and then maintained at 1–1.5% isoflurane during surgery and light-emitting diode (LED) irradiation. The reason to use isoflurane was based on the benefits of rapid induction and easy recovery from anesthesia, and isoflurane was reported being relatively safe with limited changes of blood pressure and cardiac output [21, 22]. The rats were placed in supine position. A 1-cm incision was made in the left inguinal area, and the femoral vein was carefully dissected out. For rose bengal injection (60 mg/kg; sigma- Aldrich, St. Louis, MO, USA), a polyethylene tube (PE-10; Becton, Dickinson and Company, Franklin Lakes, New Jersey, USA) was inserted into femoral vein via the dissected incision and secured with 3-0 nylon suture. Another procedure with a transverse incision in superior aspect of the sternal manubrium was made. Blunt dissection of the left CCA was performed. The CCA was laid in the ditch on LED lens, and then the CCA was fixed on the LED assembly by using suture to wrap them along the four cuts. After implantation, surgical wound was sutured, and rats were placed in prone position on the transmission coil of external controller. Then, isoflurane was decreased to 1%, and rose bengal was administrated intravenously as the photosensitizing dye. By powering on external controller, CCA was irradiated by the implanted LED (540 nm). After single irradiation with 6 mW/cm^2^ LED for 4 h, the implanted LED was removed, and the wound was sutured using 3-0 nylon. Rats were allowed to return to their cages, and food and water were provided ad libitum. All rats were included after the surgical procedures for analysis, since LED irradiation only produced local injury to common carotid artery and intravenous injection of rt-PA and mouse iPSCs did not cause severe injury to rat brain.

### Preparation of rt-PA

The rt-PA was obtained from the manufacturer (Activase, Genentech, San Fransisco, CA, USA) as lyophilized powder. The powder was mixed with sterile water in each vial to a concentration of 1 mg/mL and stored at – 80 °C before use.

### Culturing of mouse iPSCs

Mouse iPSCs (CAT# SC201A-iPSC, passage 3, System Biosciences, Mountain View, CA, USA) were used. iPSCs were cultured in 10 mL of ESGRO complete plus serum-free clonal grade medium (CAT#SF001-B, Millipore, Billerica, MA, USA) supplemented with Glycogen Synthase Kinase 3β inhibitor plus fetal bovine serum (CAT#SF012-250, Millipore, Billerica, Massachusetts, USA) in a 75 cm^2^ flask (pre-coated with 0.1% gelatin, Sigma, Merck, German) and incubated at 37 °C with 5% humidified CO_2_. The mouse iPSC cell viability was not examined before injection. The reasons were first, the regulations of culture conditions for feeder cells and growth conditions for mouse iPSCs were strictly followed according to user manual (Cat.# SCxxxA-1, System Biosciences, Mountain View, CA, USA) to ensure the highest level of cell viability. Second, we followed our previous studies [23–25] and collected passage 6–8 mouse iPSC to prepare to a final concentration of 1 × 10^6^ cells per 0.3 mL in PBS before experiment.

### In vivo detection of carotid thrombosis by animal ultrasonography

The formation and progression of thrombosis in CCA was examined at five time points including before LED irradiation and at 2 h, 24 h, 4 days and 7 days after irradiation. Rats were quickly anaesthetized with 3% isoflurane in a mixture of 30% oxygen and 70% nitrogen and maintained at 1.5% isoflurane during ultrasonography examination. B-mode and pulse-wave Doppler studies were performed using a high-resolution imaging system (Vevo 2100, Visual-Sonics, Toronto, Ontario, Canada) equipped with a 40 MHz transducer (MS 550D) from Visual-Sonics. The transverse scans of CCA were located first and then the probe was rotated 90 degrees to obtain longitudinal images. The intima-media thickness and luminal stenosis of CCA were examined by B-mode imaging. The change in the velocity of blood flow was measured by pulse-wave Doppler. Stenotic degree was calculated by [(the lumen diameter in CCA − the diameter in the narrowest site caused by thrombus)/the lumen diameter in CCA] × 100% according to our previous report (Additional file 1: Fig. S1) [20].

### Immunohistochemistry staining

The expression of inflammatory factors, Endothelin-1 (ET-1), in CCA were investigated at 7 days after LED-induced carotid thrombosis. Sections were processed using the cross sections of CCA (10 μm thickness) and stored in OCT at − 80 °C before use. Sections were handled to remove OCT with phosphate-buffered saline (PBS) for 10 min. Next, Dako EnVision®+ Dual Link System-HRP (DAB+) (CAT#K4065, Agilent Technologies, CA, USA) was used according to manufacturer’s instruction. Rabbit polyclonal antibody against ET-1 (1:200; ab117757, Abcam, Cambridge, MA, USA) was applied for 30 min, and labelled polymer (CAT#K4003, Agilent Technologies, CA, USA) was used to cover specimen for another 30 min. The specimens were then covered with substrate-chromogen solution for 5 min and after mounted with a mounting medium, entellan (CAT#107961, Merck, Germany), the specimens were cover-slipped for microscopic study.

### Evaluating the protein content of ET-1 by ELISA

At the end of each experiment (7th day), all rats were anesthetized with 3% isoflurane in a mixture of 30% oxygen and 70% nitrogen. The blood was collected from the left carotid artery in eppendorf tubes at 7 days after LED irradiation and immediately centrifuged with 3500 rpm for 10 min at 4 °C. The serum was stored at − 80 °C until analysis. Serum levels of ET-1 protein were analyzed by enzyme-linked immunosorbent assay (ELISA, BD Biosciences, MA, USA) according to manufacturer’s instructions.

### Immunoconfocal staining in carotid artery

Immunoconfocal detection of mouse stage-specific embryonic antigen 1 (SSEA1 used as iPSC marker) [26], inflammatory markers including ET-1, intercellular cell adhesion molecule-1 (ICAM-1), and interleukin-1 beta (IL-1 beta), and angiogenic trophic factors including angiopoietin-2 (AP-2), brain-derived neurotrophic factor (BDNF) and vascular endothelial growth factor (VEGF) was performed on rat CCA sections at the time points of before LED irradiation and 1 and 7 days after LED irradiation according to our previous method [24]. The CCA sections were cut at 10 μm thickness and stored in OCT at − 80 °C before use. Before staining, OCT was removed and then sections were fixed in 4% paraformaldehyde in PBS solution for 10 min. Primary antibodies against ET-1 (1:200; ab117757, Abcam, Cambridge, MA, USA), ICAM-1 (1:250, ab171123, Abcam, Cambridge, MA, USA), IL-1 beta (1:200, sc-7884, Santa Cruz Biotechnology, Santa Cruz, CA, USA), AP-2 (1:500, ab155106, Abcam, Cambridge, MA, USA), BDNF (1:500, ab108319, Abcam, Cambridge, MA, USA) and VEGF (1:200, ab46154, Abcam, Cambridge, MA, USA) were stained with donkey anti-rabbit IgG (H + L) highly cross-adsorbed secondary antibody (1:250; Catalog # A-21206, Alexa Fluor 488, Invitrogen, Carlsbad, CA, USA), except ICAM-1 with donkey anti-mouse IgG (H + L) highly cross-adsorbed secondary antibody (1:250; Catalog # A-21202, Alexa Fluor 488, Invitrogen, Carlsbad, CA, USA). Primary antibody against SSEA1 (1:200; ab16285, Abcam, Cambridge, USA) was stained with rat monoclonal [1B4B1] anti-mouse IgM mu chain secondary antibody (1:200, ab99599, Abcam, Cambridge, USA). Negative controls were performed without primary antibodies. Stained sections were mounted onto slides with mounting medium containing 4’,6-diamidino-2-phenylindole (DAPI for nuclear staining, Santa Cruz Biotechnology, Santa Cruz, CA, USA). A spot charge-coupled device colour digital camera was used to obtain immunohistochemistry images under an Olympus BX-51 microscope (Olympus Corp, Japan) and immunofluorescent images under a Leica TCS SP8X confocal laser scanning microscope (Leica Microsystem, Heidelberg, Germany) with the appropriate filters for fluorescein isothiocyanate and DAPI.

### Preparation of iRFP^+^-iPSCs

Near-infrared fluorescent protein (iRFP) [27] is a sensitive marker of cell membrane, and the production of iRFP^+^-iPSCs was done according to previous method [28]. For lentiviral production, human embryonic kidney cells 293 T (HEK 293 T; BCRC Number 60019, Bioresource Collection and Research Center, Food Industry Research and Development Institute, Hsinchu, Taiwan) was maintained in Dulbecco’s modified Eagle’s medium (DMEM, CAT#11965-092, Gibco, Thermo Fisher Scientific, Inc., Waltham, MA) containing 10% Fetal Bovine Serum (FBS, CAT#10437-028, Gibco, Thermo Fisher Scientific, Inc., Waltham, MA) and 1% penicillin/streptomycin (CAT#15140-122, Gibco, Thermo Fisher Scientific, Inc., Waltham, MA) in a humid incubator with 5% CO_2_ at 37 °C. According to the manufacturer, PolyJet™ In Vitro DNA Transfection Reagent (CAT#SL100688, SignaGen® Laboratories, Rockville, MD) was used for transfection. Lentivirus-containing medium, which was collected 24 h and 48 h after co-transfection of the *p*LV-SFFV-iRFP-P2A-Puro (CAT#DNA1046, Lmanis Life Sciences, Rochester, MN, USA) and each packaging vector (*p*CMVdeltaR8.91 and *p*MD.G; National *RNAi Core* Facility Platform, Taipei, Taiwan) into HEK 293 T cells, was centrifuged at 6000×*g* for 15 min. The supernatant was collected and stored at − 80 °C. Following overnight culture of iPSCs (1 × 10^5^ cells/12-well), the cells were infected with the supernatant containing 5 μg/mL Polybrene Infection/Transfection Reagent (CAT#TR-1003-G, Sigma-Aldrich, St. Louis, MO, USA). After infection in 2 consecutive days, the stable cell lines were selected by puromycin dihydrochloride (5 μg/mL, P9620, Sigma-Aldrich, St. Louis, MO, USA). For identification and isolation, the iRFP^+^-iPSCs were dissociated into a single-cell suspension by using ESGRO Complete Accutase (CAT#SF006, Millipore, Billerica, MA, USA), and the aggregates were removed by passing a Falcon® 5 mL Round-Bottom Polystyrene Test Tube with Cell Strainer Snap Cap (CAT#352235, Corning, Deeside, UK). The iRFP^+^-iPSCs were sorted by a flow cytometry (BD FACSAria Fusion, BD Biosciences, San Jose, CA, USA) and then, kept in the culture medium for expansion. Before immunoconfocal stain, the iRFP^+^-iPSCs were confirmed again by a BD FACSCanto™ II flow cytometry (BD FACSAria Fusion, BD Biosciences, Franklin Lakes, NJ, USA) (Additional file 2: Fig. S2).

### Immunoconfocal colocalization of iRFP^+^-iPSCs with AP-2, BDNF and VEGF

Immunoconfocal colocalization of iRFP^+^-iPSCs with AP-2, BDNF and VEGF was done in iRFP^+^-iPSCs culture using the same staining method mentioned above for carotid artery section.

### Hematoxylin and eosin (H&E) and immunoconfocal stain of 4 organs

For the detection of the possibility of iPSCs induced teratoma, 6 rats were treated with intravenous bolus injection of 1 × 10^6^ mouse iPSCs alone. The rats were given tap water ad libitum and were maintained in a temperature and humidity-controlled room on a 12-h light/dark cycle for 28 days. The rats were anesthetized with 3% isoflurane in a mixture of 30% oxygen and 70% nitrogen before euthanasia. The organs of heart, kidney, liver and lung were dissected out, frozen in powder dry ice and stored in − 80 °C freezer. Organ was cut at a thickness of 10 μm for 10 consecutive sections in each cycle with 1–3 mm interval between cycles, depending on the size of organ. The H&E stain was done according to our previous publication [29]. The immunoconfocal stain of SSEA1 was done as mentioned above.

### Statistical analysis

Sample size calculation was done by using a crude method based on law of diminishing return with the equation of *E* = total number of animals − total number of groups. After the calculation with (5 groups × 8 gerbils/group) − (5 groups) = 35, the number of 35 was more than 20, suggesting the sample size in this experiment could be more than necessary, and 8 gerbils were used for each group [30]. The experimental procedures in “LED irradiation, intravenous injection of rt-PA and mouse iPSCs”, “immunohistochemistry, immunoconfocal staining and ELISA”, and “statistical analysis” were performed by three different technical assistants who did not involve in the simple random assignment of animals to the experimental groups. Numerical sample identifiers were used during sample preparation and analysis, and the investigators were blind to the treatment. Data were analyzed with Prism 5 (GraphPad Software, La Jolla, CA, USA) and expressed as mean ± SD for continuous variables. Continuous data were compared among the groups by using one-way analysis of variance. Tukey–Kramer test was used for post-hoc comparisons. Statistical significance was established for *P* value < 0.05.

## Results

### Schema of experiment using LED irradiation

**Fig. 1:**
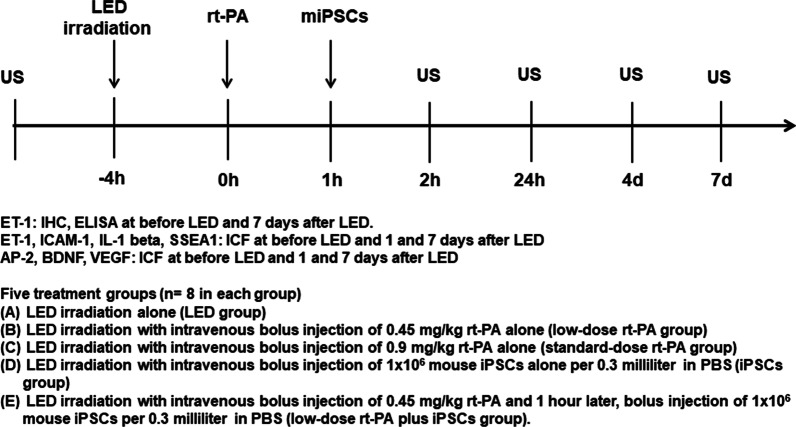
Schema of experimental procedure. Experimental procedure of the five treatment groups (*n* = 8 in each group) is demonstrated. AP-2 = angiopoietin-2; BDNF = brain-derived neurotrophic factor; ELISA = enzyme-linked immunosorbent assay; ET-1 = endothelin-1; ICAM-1 = intercellular adhesion molecule-1; ICF = immunoconfocal staining; IHC = immunohistochemistry; IL-1 beta = interleukin-1 beta; LED = light-emitting diode; miPSCs = mouse induced pluripotent stem cells; rt-PA = recombinant tissue plasminogen activator; SSEA1 = stage-specific embryonic antigen 1; US = small-animal carotid ultrasound study; VEGF = vascular endothelial growth factor. h = hour; d = day

Five treatment groups (*n* = 8 in each group) were studied including (A) LED irradiation alone (LED group), (B) LED irradiation plus intravenous bolus injection of 0.45 mg/kg rt-PA alone (low-dose rt-PA group), (C) LED irradiation plus intravenous bolus injection of 0.9 mg/kg rt-PA alone (standard-dose rt-PA group), (D) LED irradiation plus intravenous bolus injection of 1 × 10^6^ mouse iPSCs alone per 0.3 mL in PBS (iPSCs group), and (E) LED irradiation plus intravenous bolus injection of 0.45 mg/kg rt-PA and 1 h later, bolus injection of 1 × 10^6^ mouse iPSCs per 0.3 mL in PBS (low-dose rt-PA plus iPSCs group). The experimental procedure is presented in Fig. 1.

### CCA thrombotic occlusion model after LED irradiation alone

The CCAs were examined using small-animal ultrasound system at each time point to show the temporal change of carotid thrombosis after LED irradiation (Additional file 3: Fig. S3). The ultrasound showed no CCA obstruction before LED irradiation. After single LED irradiation with 6 mW/cm^2^ for 4 h, CCAs were completely occluded and no Doppler signal could be detected from 2 h, 24 h, 4 days to 7 days compared to control. The complete thrombus formation from 2 h after irradiation suggested the success of the present thrombotic occlusion model.

### CCA thrombotic occlusion model after rt-PA and/or iPSCs treatment

**Fig. 2:**
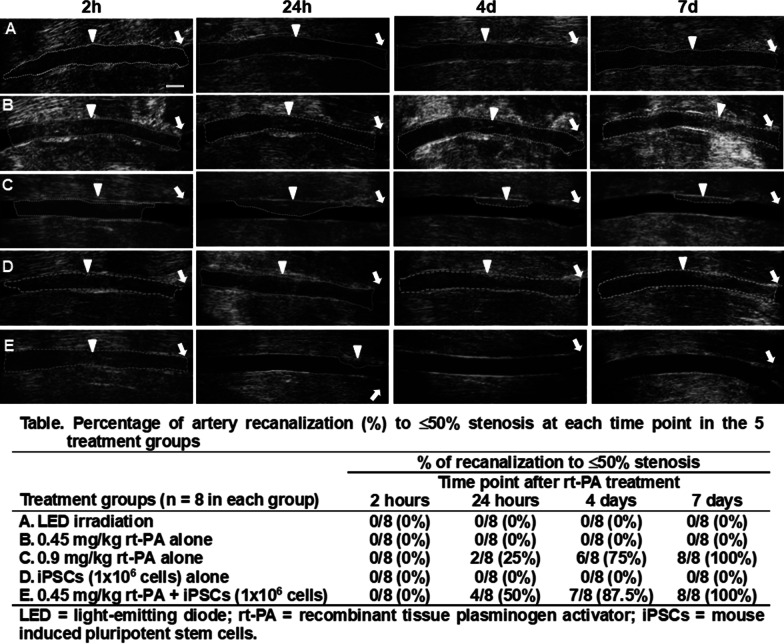
Ultrasound studies of common carotid artery (CCA) and the ratio of recanalization to ≤ 50% stenosis in the 5 treatment groups at 4 time points after 6 mW/cm^2^ LED irradiation for 4 h. CCD lumen is completely occluded at **A**, **B** and **D** groups from 2 h, 24 h, 4 days to 7 days. However, CCA lumen is occluded at 2 h but progressively recanalized from 24 h, 4 days to 7 days at **C** and **E** groups. **A**  LED irradiation alone (LED group), **B** LED irradiation plus intravenous bolus injection of 0.45 mg/kg rt-PA alone (0.45 mg/kg rt-PA group), **C**  LED irradiation plus intravenous bolus injection of 0.9 mg/kg rt-PA alone (0.9 mg/kg rt-PA group), **D** LED irradiation plus intravenous bolus injection of 1 × 10^6^ iPSCs alone (iPSCs group), **E** LED irradiation plus intravenous bolus injection of 0.45 mg/kg rt-PA and 1 h later, bolus injection of 1 × 10^6^ iPSCs (0.45 mg/kg rt-PA plus iPSCs group). LED = light-emitting diode; n = 8 in each group. Arrow indicates carotid bifurcation. Arrowhead and dot-line indicate the stenosis segment. h = hour; d = day. Scale bar in A (2 h) = 1 mm

The CCAs were examined by small-animal ultrasound system in the 5 groups at each time point (Fig. 2 and Table). In all groups, complete thrombotic occlusion could be observed at 2 h after irradiation. The group A (LED irradiation alone) had persistent occlusion from 2 h to 7 days. Bolus administration of 0.45 mg/kg rt-PA alone (group B) or 1 × 10^6^ iPSCs alone (group D) caused partial recanalization of CCA thrombotic occlusion from 2 h to 7 days, but the percentage of recanalization to ≤ 50% stenosis was similar to group A. The administration of 0.9 mg/kg rt-PA alone (group C) induced good recanalization with resulting stenotic degree ≤ 50% seen from 24 h, 4 days to 7 days (recanalization rate with ≤ 50% stenosis: 25%, 75% and 100%, respectively). If combined administration of 0.45 mg/kg rt-PA plus 1 × 10^6^ iPSCs (group E) was performed, there was a better recanalization rate with stenotic degree ≤ 50% (50%, 87.5% and 100% at 24 h, 4 days and 7 days, respectively) compared to 0.9 mg/kg rt-PA alone (group C).

### ET-1 immunostaining at 7 days in 5 treatment groups

Immunohistochemical examination of ET-1 at 7 days (Additional file 4: Fig. S4) showed there was normal architecture of CCA artery wall with mild ET-1 immunoreactivity before LED irradiation. At 7 days after LED irradiation, thrombosis could be found inside CCA lumen in the groups of LED alone, 0.45 mg/kg rt-PA alone and iPSCs alone, and endothelial membrane was severely injured with less injury in the iPSCs alone group. However, thrombus was not present inside CCA lumen and endothelial membrane was relatively preserved in the 0.9 mg/kg rt-PA alone and 0.45 mg/kg rt-PA plus iPSCs groups. ET-1 immunoreactivity was induced remarkably in thrombus and in LED-irradiated luminal wall of CCA but was less induced in the luminal wall of iPSCs treatment groups. ET-1 immunoreactivity was slightly induced to near the level of before irradiation in 0.45 mg/kg rt-PA plus iPSCs group.

### ET-1 level in arterial blood at 7 days in 5 treatment groups

ET-1 level in CCA blood was examined by ELISA at 7 days after 6 mW/cm^2^ LED irradiation for 4 h in each treatment group (Additional file 5: Fig. S5). ET-1 levels were significantly higher in LED alone group (9.405 pg/mL), 0.45 mg/kg rt-PA alone group (9.410 pg/mL), and iPSCs alone group (9.458 pg/mL) compared to the levels of before irradiation (1.480 pg/mL) and 0.9 mg/kg alone (2.116 pg/mL) and 0.45 mg/kg plus iPSCs groups (1.486 pg/mL) (*P* < 0.01 in each comparison). Significant reductions were observed in 0.9 mg/kg rt-PA alone group (2.116 pg/mL) compared to 0.45 mg/kg rt-PA alone group (9.410 pg/mL) (*P* < 0.01). The ET-1 level in 0.45 mg/kg rt-PA plus iPSCs group (1.486 mg/mL) was similar to that before LED irradiation (1.480 pg/mL) but showed a significant reduction compared to that in 0.9 mg/kg rt-PA alone group (2.116 pg/mL) (*P* < 0.05).

### Single immunoconfocal localization of DAPI and SSEA1 with ET-1, ICAM-1, IL-1 beta, AP-2, BDNF, VEGF

**Fig. 3:**
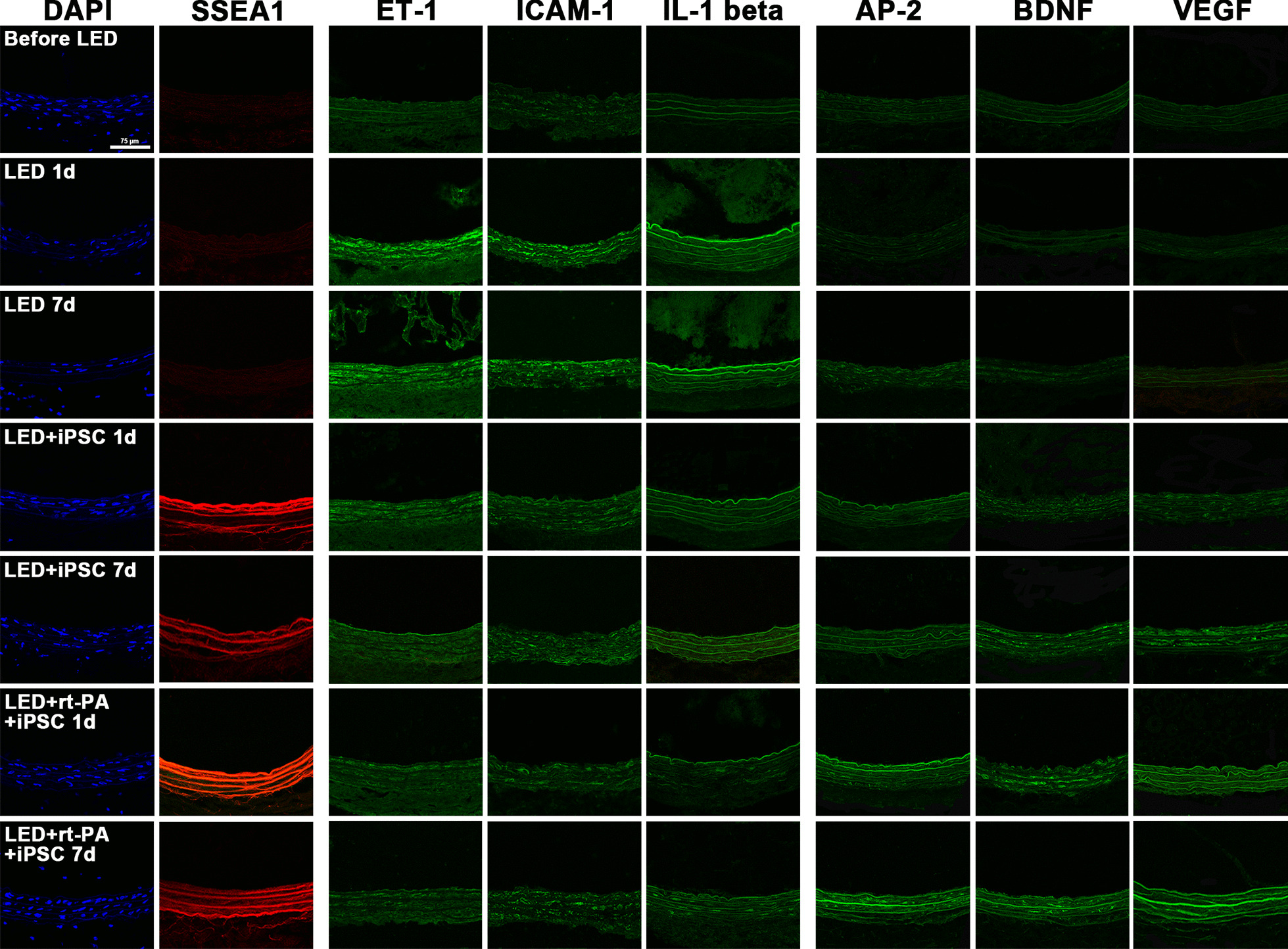
Single immunoconfocal localization of DAPI (blue color) and SSEA1 (red color) with ET-1, ICAM-1, IL-1 beta, AP-2, BDNF, and VEGF (green color) in carotid artery wall. DAPI can be seen before LED irradiation and at 1 day and 7 days after LED irradiation. SSEA1 is not visible before LED irradiation and at 1 day and 7 days in LED alone group, but is strongly visible at 1 day and 7 days after iPSC treatment. ET-1, ICAM-1, and IL-1 beta are rarely seen in CCA artery wall before LED irradiation. However, ET-1, ICAM-1, and IL-1 beta are stained strongly at 1 day and 7 days in LED alone group, but their immunofluorescence is reduced after iPSCs treatment. AP-2, BDNF and VEGF are mildly expressed in CCA artery wall before LED irradiation. However, the expressions of AP-2, BDNF and VEGF are reduced in LED alone group at 1 day and 7 days. After iPSCs treatment, the expressions of AP-2, BDNF and VEGF are increased. AP-2 = angiopoietin-2; BDNF = brain-derived neurotrophic factor; DAPI = 4’,6-diamidino-2-phenylindole; ET-1 = endothelin-1; ICAM-1 = intercellular adhesion molecule-1; IL-1 = interleukin 1 beta; iPSCs = mouse induced pleuripotent stem cells; LED = light-emitting diode; SSEA1 = stage-specific embryonic antigen 1; VEGF = vascular endothelial growth factor

Immunoconfocal localization of DAPI and SSEA1 with ET-1, ICAM-1, IL-1 beta, AP-2, BDNF, and VEGF was examined in CCA artery before LED irradiation and at 1 day and 7 days after LED irradiation in LED alone, LED with iPSCs, and LED with iPSCs plus 0.45 mg/kg rt-PA groups (Fig. 3). DAPI could be visible in the artery wall of CCA before LED irradiation but the number of DAPI-stained cells was reduced at 1 day and 7 days after LED irradiation in every group. SSEA1 was not visible in the groups of before LED and LED alone. However, SSEA1 expression was clearly visible at 1 day but less visible at 7 days after iPSCs treatment compared to before LED. ET-1, ICAM-1, and IL-1 beta were rarely expressed in CCA artery before LED irradiation. However, ET-1, ICAM-1, and IL-1 beta were strongly expressed at 1 day, and the expression was slightly reduced at 7 days in LED alone group. After iPSCs treatment, the expression of ET-1, ICAM-1, and IL-1 beta were recovered to the levels near before LED.

**Fig. 4:**
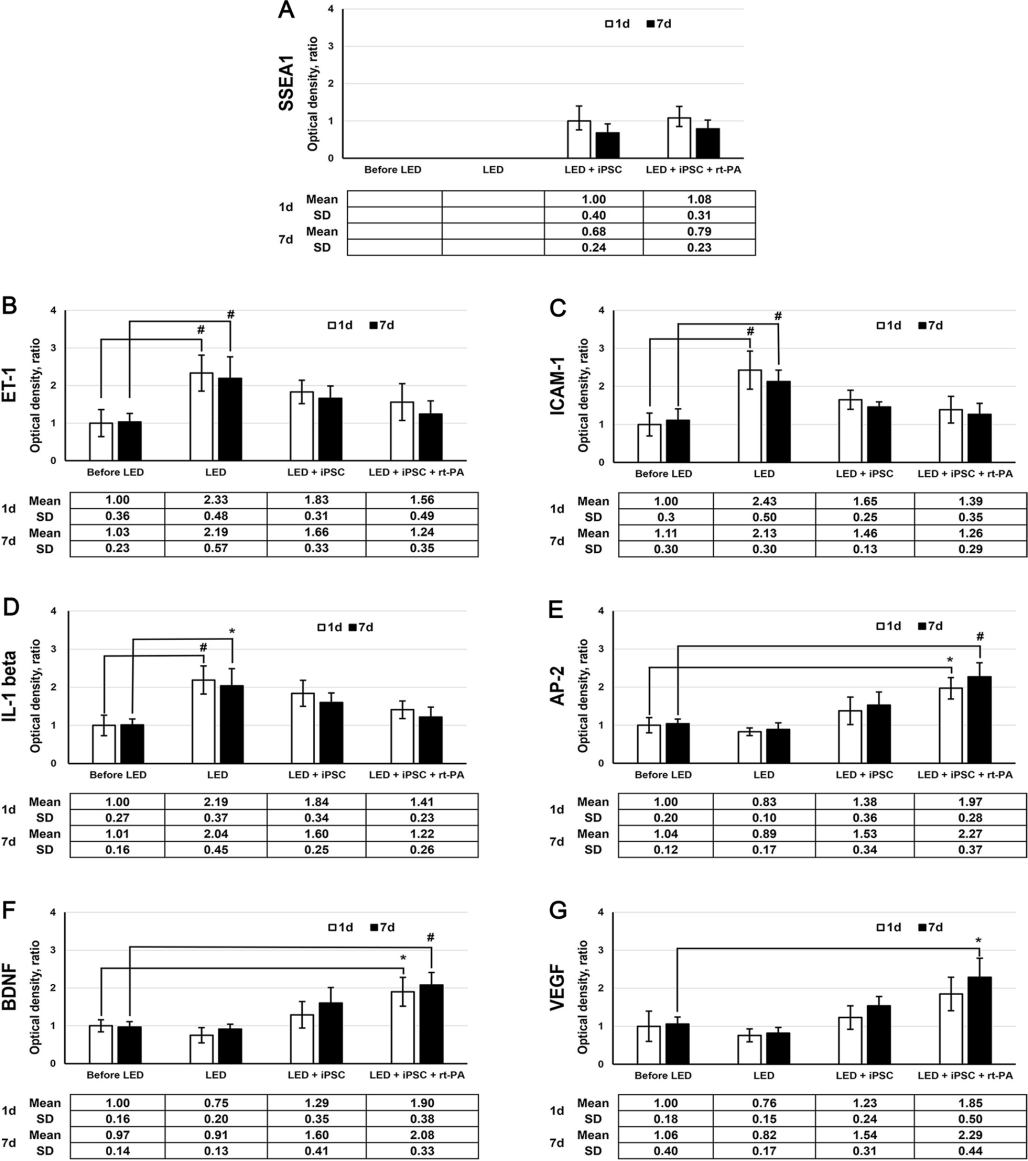
Statistical results of the optical density of single immunoconfocal staining of SSEA1, ET-1, ICAM-1, IL-1 beta, AP-2, BDNF and VEGF. One-way analysis of variance with Tukey–Kramer test for post-hoc comparisons is used for multiple comparison of means. *P < 0.05, ^#^*P* < 0.01

AP-2, BDNF and VEGF could be mildly expressed in CCA artery before LED irradiation. However, the expressions of AP-2, BDNF and VEGF were reduced in LED alone group at 1 day and 7 days. After iPSCs treatment, the expressions of AP-2, BDNF and VEGF were recovered, and they were improved significantly to above the levels of before LED in iPSCs plus rt-PA group at 1 day and 7 days (Fig. 3). The statistical results of the optical density of SSEA1, ET-1, ICAM-1, IL-1 beta, AP-2, BDNF and VEGF expressions are presented in Fig. 4.

### Immunoconfocal colocalization of DAPI, ET-1, IL-1 beta, AP-2, BDNF, VEGF with SSEA1

**Fig. 5:**
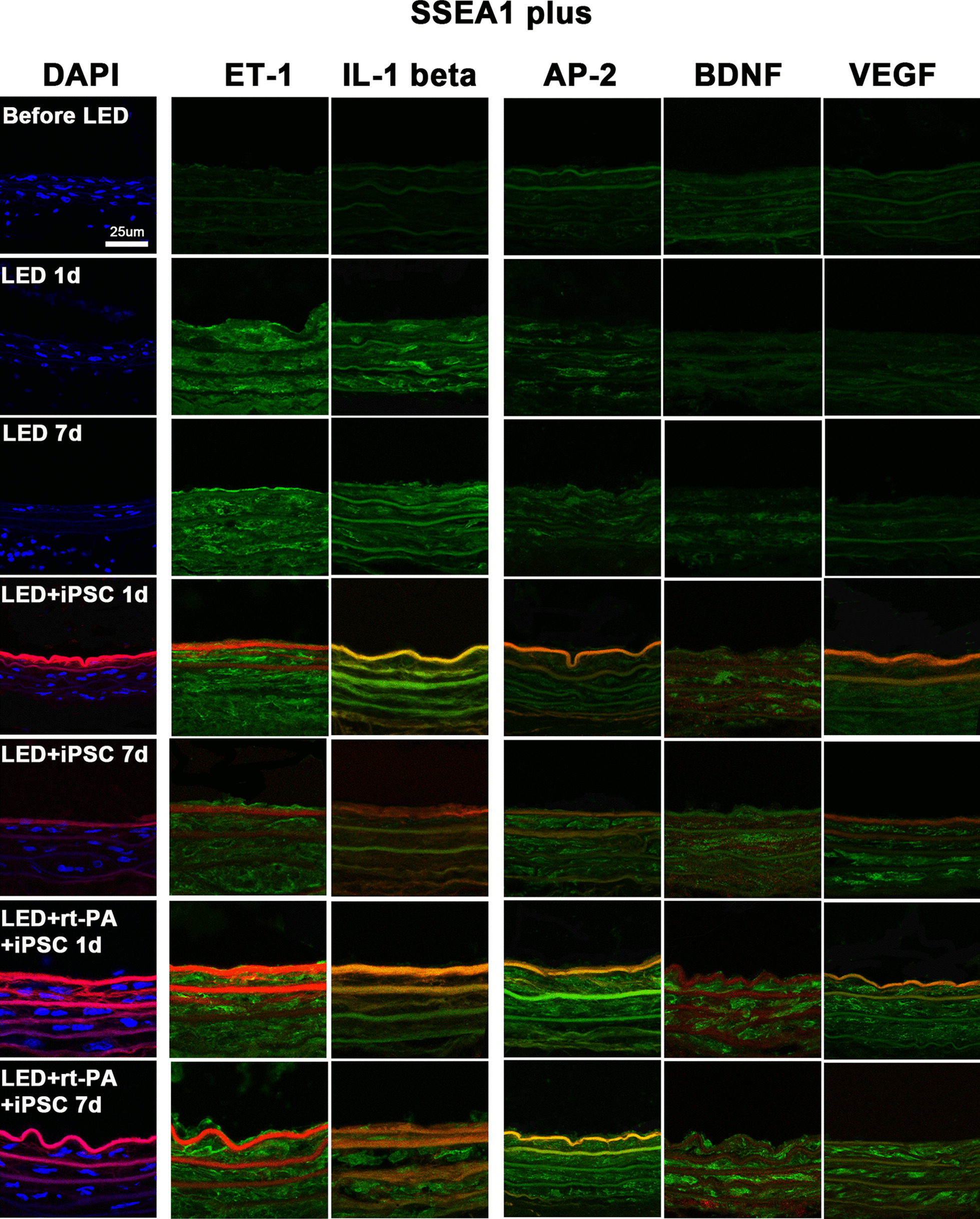
Immunoconfocal colocalization of SSEA1 (red color) with DAPI (blue color), and ET-1, ICAM-1, IL-1 beta, AP-2, BDNF, and VEGF (green color) in CCA artery wall. SSEA1 is not visible in the groups of before LED and LED alone but is strongly expressed after iPSCs treatment at 1 day and 7 days. ET-1 and IL-1 beta are rarely expressed before LED irradiation. However, they show strong expressions in LED alone group at 1 day and 7 days, and the expressions are reduced after iPSCs treatment. AP-2, BDNF and VEGF are lightly visible in before LED group and reduced in LED alone group at 1 day and 7 days. However, AP-2, BDNF and VEGF are increased after iPSCs treatment. ICAM-1 is not colocalized with SSEA1 due to the conflict of secondary antibodies. AP-2 = angiopoietin-2; BDNF = brain-derived neurotrophic factor; DAPI = 4’,6-diamidino-2-phenylindole; ET-1 = endothelin-1; ICAM-1 = intercellular adhesion molecule-1; IL-1 = interleukin 1 beta; iPSCs = mouse induced pleuripotent stem cells; LED = light-emitting diode; SSEA1 = stage-specific embryonic antigen 1; VEGF = vascular endothelial growth factor

The colocalization of SSEA1 with DAPI, ET-1, IL-1 beta, AP-2, BDNF and VEGF (Fig. 5) were studied. ICAM-1 was not colocalized with SSEA1 due to the conflict of secondary antibodies. SSEA1 expression was not visible in the groups of before LED and LED alone at 1 day and 7 days. However, SSEA1 was strongly expressed in the LED groups with iPSCs treatment at 1 day but less expressed at 7 days. ET-1 and IL-1 beta were rarely expressed in before LED group. However, ET-1 and IL-1 beta showed strong expressions in LED alone group at 1 day and 7 days, but the expressions were reduced after iPSCs treatment.

The expressions of AP-2, BDNF and VEGF were lightly visible in before LED group and were reduced in LED alone group at 1 day and 7 days. However, the expressions of AP-2, BDNF and VEGF were increased after iPSCs treatment especially in the LED group with low-dose rt-PA plus iPSCs treatment.

### Identification of iRFP^+^-iPSCs by flow cytometry

After iRFP^+^-iPSCs were generated by infection with lentiviral product, flow cytometry sorted iRFP^+^-iPSCs, which proved that iPSCs were successfully infected with lentiviral product and could express iRFP protein. As shown in Additional file 2: Fig. S2A, after iPSCs were infected with lentiviral product, the percentage of iRFP protein expression in iPSCs was 41.3%. To purify the iRFP^+^-iPSCs, we gated the near-infrared fluorescence (Alexa 680) and collected them using flow cytometry. After flow cytometry sorting, the percentage of iRFP protein expression in iPSCs was 96.0% (Additional file 2: Fig. S2B). The results suggested that sorting could improve the purity of iRFP^+^-iPSCs. After sorting and amplification of iRFP^+^-iPSCs, the percentage of iRFP^+^-iPSCs was 98.5% (Additional file 2: Fig. S2C). The results indicated that the percentage of iRFP^+^-iPSCs did not change much after sorting and culture, indicating that the lentivirus-iRFP plasmid could be used to label iPSCs.

### Immunoconfocal staining in iRFP^+^-iPSCs culture

**Fig. 6:**
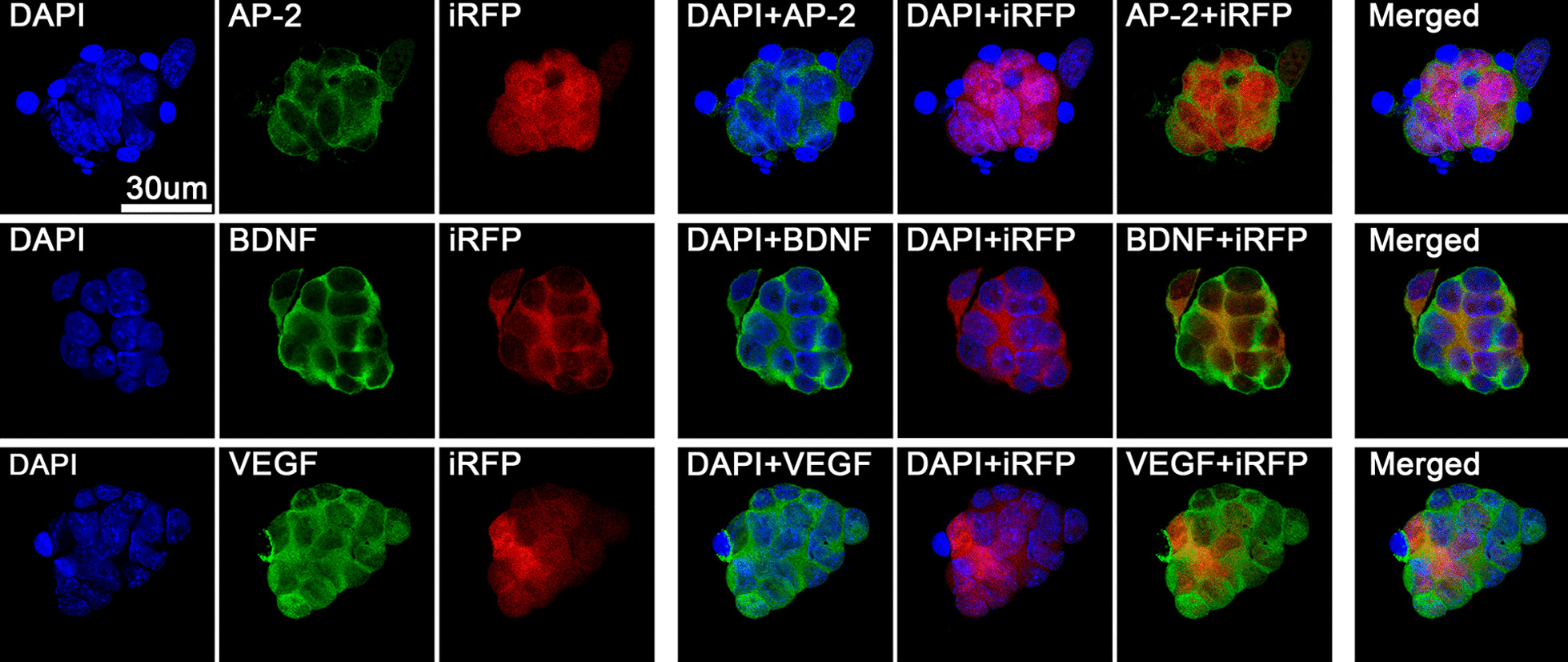
Immunoconfocal colocalization of iRFP^+^-iPSCs and DAPI with AP-2, BDNF or VEGF. AP-2, BDNF and VEGF are colocalized with DAPI in iRFP^+^-iPSCs. AP-2 = angiopoietin-2; BDNF = brain-derived neurotrophic factor; DAPI = 4’,6-diamidino-2-phenylindole; iRFP = near-infrared fluorescent protein; VEGF = vascular endothelial growth factor

Immunoconfocal colocalization of iRFP^+^-iPSCs and DAPI with AP-2, BDNF or VEGF showed iRFP^+^-iPSCs could be stained with AP-2, BDNF and VEGF (Fig. 6).

### H&E stain and immunoconfocal stain for the detection of teratoma

**Fig. 7:**
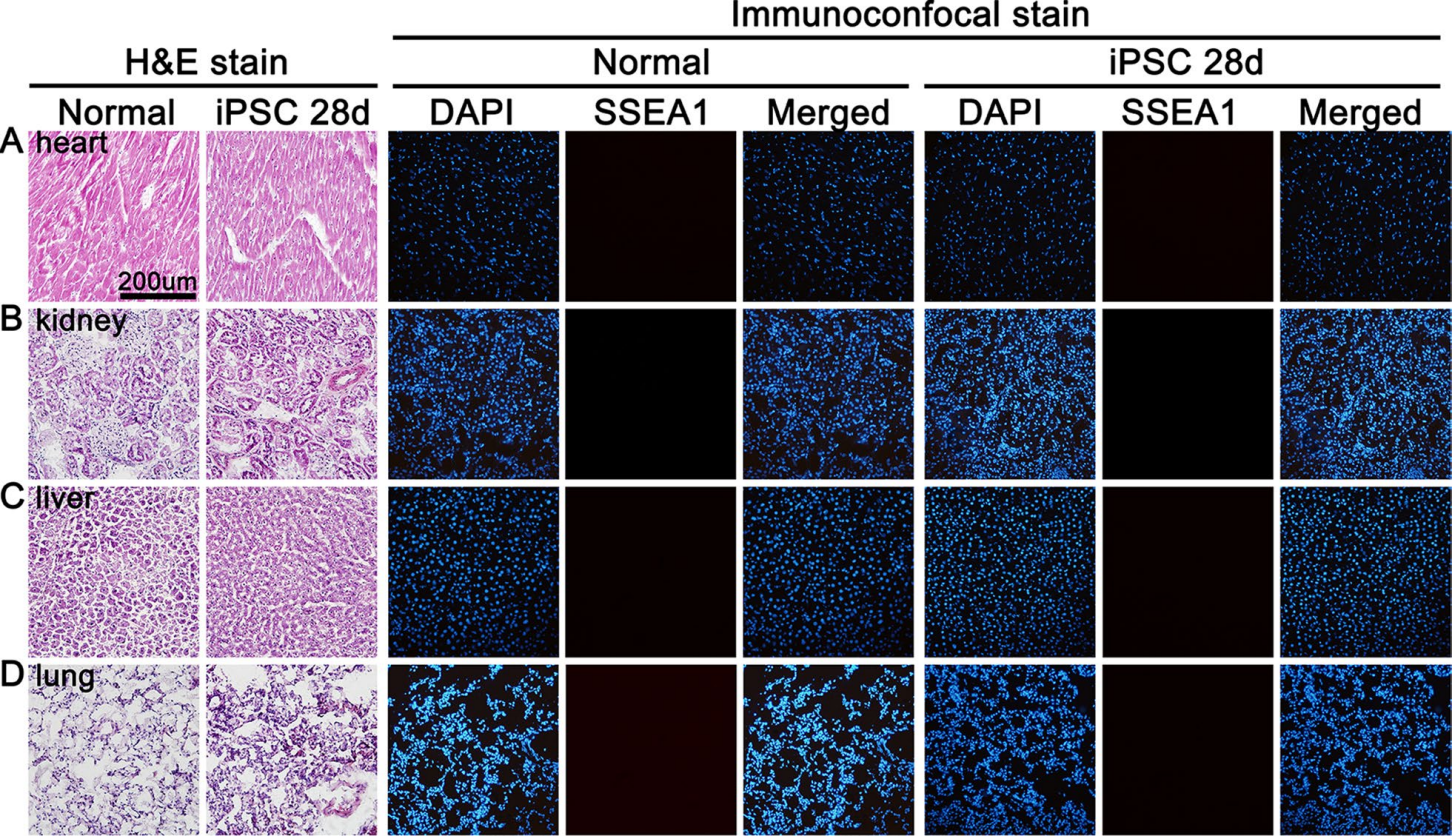
Hematoxylin and eosin (H&E) and immunoconfocal stain of heart, kidney, liver and lung at 28 days after intravenous iPSC treatment. H&E stain shows no abnormal cells can be found and immunoconfocal stain shows SSEA1-positive immunoreactivity is not seen in heart, kidney, liver and lung compared to normal. DAPI = 4’,6-diamidino-2-phenylindole; iPSCs = mouse induced pleuripotent stem cells; SSEA1 = stage-specific embryonic antigen 1

H&E stain and immunoconfocal stain showed there was no abnormal cells in H&E stain and SSEA1 immunoreactivity was not seen in heart, kidney, liver and lung (Fig. 7).

## Discussion

The present study used half dose of standard-dose rt-PA, which is lower than the clinical practice of low-dose rt-PA (0.6–0.9 mg/kg). We found 0.45 mg/kg rt-PA alone caused no recanalization in rat CCA thrombosis compared to full recanalization with 0.9 mg/kg rt-PA. This result of no recanalization using 0.45 mg/kg rt-PA is similar to our previous study in rats [19] and may explain the reason why the use of half-dose rt-PA is not recommended in clinical practice. However, the present study showed the adjuvant treatment with iPSCs could improve the recanalization effect of 0.45 mg/kg rt-PA remarkably to the condition better than 0.9 mg/kg rt-PA treatment. Also, compared to our previous study using focused ultrasound and a neuroprotectant as adjuvants to 0.45 mg/kg rt-PA [19], iPSCs can produce a better recanalization effect when treated with 0.45 mg/kg rt-PA. The present study may suggest the dose of rt-PA can be further reduced if adjuvant regimen is used such as stem cell to obtain similar thrombolytic effect but less side effects. The present study also suggests that iPSCs may act through the up-regulation of angiogenic trophic factors, AP-2, BDNF, and VEGF, to stabilize the endothelial integrity and the down-regulation of inflammatory factors, ET-1, ICAM-1 and IL-1 beta. It is possible that through these mechanisms, iPSCs can help to improve the thrombolytic effect.

ET-1 is produced in endothelial cells and is the leading molecule that regulates vascular function [31]. ET-1 is secreted by endothelial cells during hypoxia [32, 33] and is an aggravating factor of cardiovascular diseases under endothelial dysfunction [31]. ICAM-1 is an adhesion molecule related to vascular diseases. The dysfunction of endothelial cells may promote vascular inflammation by expressing surface adhesion molecules including ICAM-1 [34]. IL-1 beta is produced by endothelial cells [35] and plays a critical role in inflammation process [36]. The release of IL-1 beta by injured cells may stimulate endothelial cells to secrete chemokines and increases the expression of vascular adhesion molecules [37].

Blockade of ET-1 may provide neuroprotection to hippocampal cells through the modulation of BDNF system [38], and ET-1 may stimulate the production of BDNF in cultured rat astrocytes [39]. Clinically, elevated ET-1 levels are associated with severe disease course, while AP-2 level is negatively correlated with severity in adult hemorrhagic fever patients [40]. IL-1 beta may cause neurons at risk by interfering with BDNF signaling [41], and BDNF can attenuate the IL-1 beta-induced ICAM-1 mRNA and protein expression [42]. IL-1 beta can also stimulate the production of BDNF at the mRNA and protein levels in an IL-1 receptor dependent fashion [43]. AP-2 and BDNF can be up-regulated in the infarcted cortices grafted with human umbilical mesenchymal stem cells [44], and the expression of AP-2 at sites of vascular remodeling can initiate neovascularization [45]. AP-1 can upregulate the expression of intracellular IL-1 beta [46] and AP-2, a functional antagonist of AP-1 [47], may reverse the expression of IL-1 beta. ICAM-1 is involved in the regulation of angiopoietin expression during oxygen-driven pathologic angiogenesis [48]. Pro-angiogenic therapy using VEGF may be useful in ischemic diseases such as stroke, myocardial ischemia and coronary artery disease [49]. VEGF and IL‑1 act inversely as the risk factors in the treatment of thromboangiitis obliterans by revascularization [50], and IL-1 may induce VEGF production in chondrocytes through distinct signaling pathways [51]. VEGF has the ability to increase ICAM-1 expression in the retinal vasculature and ICAM-1 can mediate VEGF-induced vascular permeability and nonperfusion [52]. In ET-1- treated MSCs, expression of AP-2 and VEGF were increased compared to control groups [53]. The above data may suggest the interaction between inflammatory factors and angiogenic trophic factors can be involved in the pathogenesis of various diseases such as artery thrombosis.

Mouse iPSCs has been reported to induce teratoma formation with hematoxylin and eosin staining at 28 days after subcutaneous injection of 1 × 10^6^ iPSCs into the dorsal flank of SCID mice (User manual of Human and Mouse iPS Cell Lines, System Biosciences, Mountain View, CA, USA). It has been reported that teratoma formation assay is an effective method to evaluate the pluripotency of iPSCs by injection and transplantation of iPSCs into immunodeficient mice at the gastrocnemius muscle [54]. The present study used intravenous injection of iPSCs, and our H&E staining did not find any abnormal cells and immunoconfocal study did not find SSEA1-positive cells in heart, kidney, liver and lung at 28 days (Fig. 7). However, it is possible that a larger dose of iPSC and/or a longer period of observation may be needed to detect teratoma.

The reason why we used iPSCs is based on the following reasons. First, previous reviews [55–57] have demonstrated that in clinical trials, stem cell could help the recovery of neuronal function and improvement of neurological outcome in either acute or chronic stroke. Human iPSC-derived long-term neuroepithelial-like stem cells can differentiate to form functional cortical neurons when transplanted into stroke-injured cortex [58]. Second, iPSCs have pluripotency comparable to that of embryonic stem cells and are found to improve motor function, reduce infarction size, attenuate inflammation cytokines, and mediate neuroprotection after ischemic injury [59]. Third, patient-derived iPSCs can be used in disease modeling and drug screening studies, and iPSCs have the potential in rejection-tolerance personalized replacement therapy [60]. Fourth, iPSCs have the ability to generate cellular products for autologous grafts and are not followed by a graft versus-host response [61]. Fifth, although iPSC-derived cells have been used in various research, the ability of iPSC to successfully differentiate into endothelial cells is mostly in in vitro studies [62, 63]. The present study attempted to use the iPSCs with no differentiation for this preliminary study to identify if the pluripotency of iPSCs can be effective in the stabilization of carotid endothelial integrity.

The reason why we used 1 × 10^6^ mouse iPSCs but not higher concentration is based on our previous studies. We found that the concentration of 1 × 10^6^ was effective for human amniotic fluid stem cell transplantation on bladder function in many different animal models including rat models with diabetes [23], spinal cord injury [24], atherosclerosis-induced chronic bladder ischemia [64], middle cerebral artery occlusion [25] and pelvic nerve transection [65]. However, it is possible that higher concentration may have better thrombolytic effect when added to 0.45 mg/kg rt-PA after LED irradiation.

Our previous study [20] demonstrated that rose bengal plus LED irradiation may induce artery endothelium injury and cause platelet adhesion and thrombus formation. The present study showed the integrity of endothelial membrane was impaired, and ET-1, ICAM-1 and IL-1 beta were induced in the artery wall with the blood level of ET-1 elevated along with the formation of luminal thrombosis after administration of rose bengal plus LED irradiation. However, iPSCs treatment can attenuate these inflammatory markers, reduce the blood level of ET-1 and preserve the integrity of endothelial membrane in acute artery thrombosis.

The present study has some limitations. First, we used only single injection of iPSCs and it is possible that multiple injections may give better effect. However, single injection is the most convenient way of treatment in clinical settings, and our previous studies [23–25, 64, 65] all used single injection of stem cell and had obtained good effect. Second, since the present study was designed based on clinical scenario, we did not examine the detailed mechanism as to how iPSCs can improve thrombolytic effect. However, we examined the expressions of ET-1, ICAM-1, IL-1 beta, AP-2, BDNF and VEGF and found ET-1, ICAM-1 and IL-1 beta were down-regulated but AP-2, BDNF and VEGF were up-regulated after iPSCs treatment. It is possible that the pleuripotent effect of iPSCs may also include anti-inflammation and secretion of trophic factors, which may help to improve thrombolytic effect. Third, we did not examine if there was intracerebral bleeding. However, our previous study [19] has shown even the dose of 0.9 mg/kg rt-PA did not cause visible cerebral hemorrhage on rat brain magnetic resonance images. Fourth, we used mouse iPSCs in the acute carotid thrombotic occlusion in rat but not in mouse. There are several reasons including (1) the technology of generating iPSCs has not been widely applied to other species other than mouse and human [66]; (2) although rat iPSCs have been established recently, reports of their use and properties are still limited [67]; (3) rat iPSCs are not easy to purchase commercially from industries; (4) our LED-induced acute carotid thrombotic occlusion model is established in rat but not in mouse.

## Conclusion

The present study is the first to indicate iPSCs can help to reduce the dose of rt-PA and improve the thrombolytic effect of 0.45 mg/kg rt-PA by acting through reducing the expressions of ET-1, ICAM-1 and IL-1 beta but inducing the expressions of AP-2, BDNF and VEGF to stabilize the endothelial integrity. It is likely there is a potential role of stem cell in acute thrombolytic treatment.

## Supplementary Information

**Additional file 1: Fig. S1**. The calculation method of stenotic degree. D = diameter.

**Additional file 2: Fig. S2.** The percentage of iRFP expression in iPSCs with or without infection with lentiviral product is determinied by flow cytometery. (A) Before flow cytometery sorting, (B) after flow cytometery sorting, and (C) after flow cytometery sorting and amplification

**Additional file 3: Fig. S3**. Ultrasound studies of common carotid artery (CCA) at 5 time points before and after single LED irradiation with 6  mW/cm^2^ for 4 hours. (A) Before LED irradiation, there is Doppler flow (red color inside the CCA lumen). (B-E) After LED irradiation, there is no Doppler flow seen in CCA lumen and no recanalization of the occluded CCA from 2 hours, 24 hours, 4 days to 7 days. Arrow indicates the carotid bifurcation, and arrowhead indicates the stenosis segment. LED = light-emitting diode; h = hour; d = day; Scale bar in A = 1 mm.

**Additional file 4: Fig. S4**. Immunohistochemical staining of endothelin-1 (ET-1) in common carotid artery (CCA) at 7 days after single 6 mW/cm^2^ LED irradiation for 4 hours. Thrombus formation can be found inside CCA lumen at B, C and E. Endothelial membrane of CCA is damaged at B and C but less injured at E. However, endothelial membrane of CCA is relatively preserved at D and F. ET-1 immunoreactivity is induced significantly in LED-irradiated luminal wall of CCA at B, C and E. ET-1 immunoreactivity is less expressed in the group with iPSCs treatment (D) and is near the level of before LED irradiation (A) at F. A = before LED irradiation, B = LED irradiation alone (LED group), C = LED irradiation plus intravenous bolus injection of 0.45 mg/kg rt-PA alone (0.45 mg/kg rt-PA group), D = LED irradiation plus intravenous bolus injection of 0.9 mg/kg rt-PA alone (0.9 mg/kg rt-PA group), E = LED irradiation plus intravenous bolus injection of 1×106 iPSCs alone (iPSCs group), F = LED irradiation plus intravenous bolus injection of 0.45 mg/kg rt-PA and 1 hour later, bolus injection of 1×106 iPSCs (0.45 mg/kg rt-PA plus iPSCs group). LED = light-emitting diode; n = 8 in each group. Arrows indicate the endothelial membrane of CCA. Scale bar in A = 100 μm. One-way analysis of variance with Tukey–Kramer test for post-hoc comparisons is used for multiple comparison of means. *P < 0.05, ^#^*P* < 0.01.

**Additional file 5: Fig. S5**. Endothelin-1 (ET-1) level is examined using common carotid artery blood by ELISA at 7 days after single 6 mW/cm^2^ LED irradiation for 4 hours in each treatment group. ET-1 levels are significantly higher at B (9.405 pg/mL), C (9.410 pg/mL), and E (9.458 pg/mL) compared to A (1.480 pg/mL), D (2.116 pg/mL), and F (1.486 pg/mL) (*P* < 0.01). There is a significant reduction at D (2.116 pg/mL) compared to C (9.410 pg/mL) (*P* < 0.01). The ET-1 level at F (1.486 mg/mL) is significantly reduced, similar to that before LED irradiation (A, 1.480 pg/mL) but much lower than that at D (2.116 pg/mL) (*P* < 0.05). A = before LED irradiation, B = LED irradiation alone (LED group), C = LED irradiation plus intravenous bolus injection of 0.45 mg/kg rt-PA alone (0.45 mg/kg rt-PA group), D = LED irradiation plus intravenous bolus injection of 0.9 mg/kg rt-PA alone (0.9 mg/kg rt-PA group), E = LED irradiation plus intravenous bolus injection of 1×106 iPSCs alone (iPSCs group), F = LED irradiation plus intravenous bolus injection of 0.45 mg/kg rt-PA and 1 hour later, bolus injection of 1×106 iPSCs (0.45 mg/kg rt-PA plus iPSCs group). LED = light-emitting diode; n = 8 in each group. *P < 0.05, D vs. A group; **P < 0.01 B, C and E vs. A, D and F groups; ^#^*P* < 0.05, D vs. F group. One-way analysis of variance with Tukey–Kramer test for post-hoc comparisons is used for multiple comparison of means.

## Data Availability

The datasets used and/or analysed during the current study are available from the corresponding author on reasonable request.

## Abbreviations

AP-2: Angiopoietin-2
BDNF: Brain-derived neurotrophic factor
CCA: Common carotid artery
DAPI: 4′,6-Diamidino-2-phenylindole
ET-1: Endothelin-1
ICAM-1: Intercellular adhesion molecule-1
IL-1: Interleukin-1 beta
iPSCs: Induced pleuripotent stem cells
iRFP^+^-iPSCs: Near-infrared fluorescent protein positive iPSCs
LED: Light-emitting diode
rt-PA: Recombinant tissue plasminogen activator
SSEA1: Stage-specific embryonic antigen 1
VEGF: Vascular endothelial growth factor
