# Supplementary figures and images for: Induced pluripotent stem cells can improve thrombolytic effect of low-dose rt-PA after acute carotid thrombosis in rat
Source: Stem Cell Res Ther. 2021 Oct 21;12:549. doi: 10.1186/s13287-021-02615-z (PMC8532293; doi:10.1186/s13287-021-02615-z)

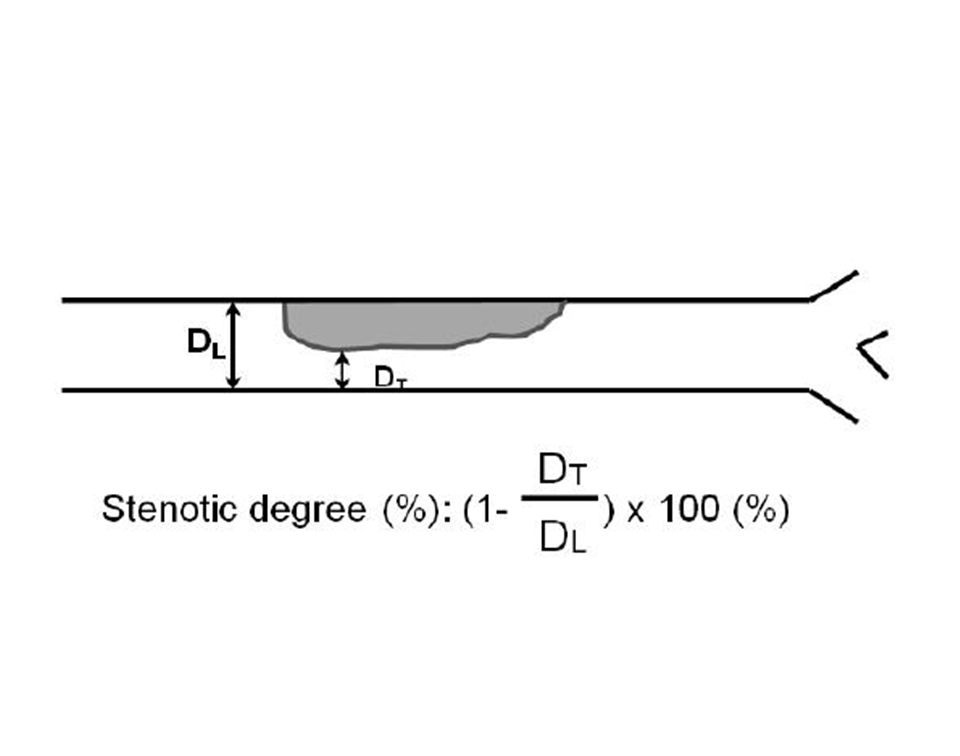

Supplement: Supplementary file 1 — Additional file 1: Fig. S1. The calculation method of stenotic degree. D = diameter. [file 13287_2021_2615_MOESM1_ESM.tif]

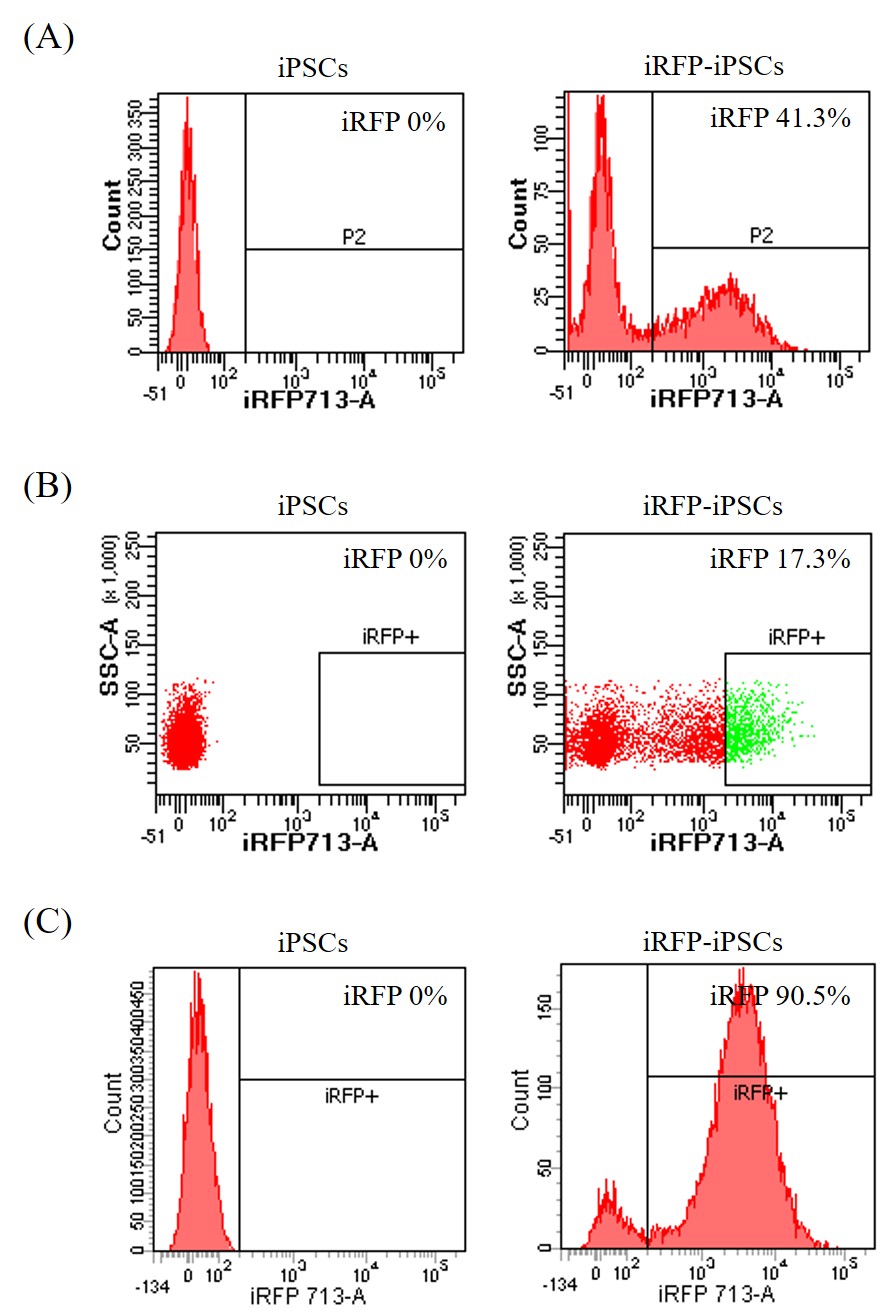

Supplement: Supplementary file 2 — Additional file 2: Fig. S2. The percentage of iRFP expression in iPSCs with or without infection with lentiviral product is determinied by flow cytometery. (A) Before flow cytometery sorting, (B) after flow cytometery sorting, and (C) after flow cytometery sorting and amplification [file 13287_2021_2615_MOESM2_ESM.tif]

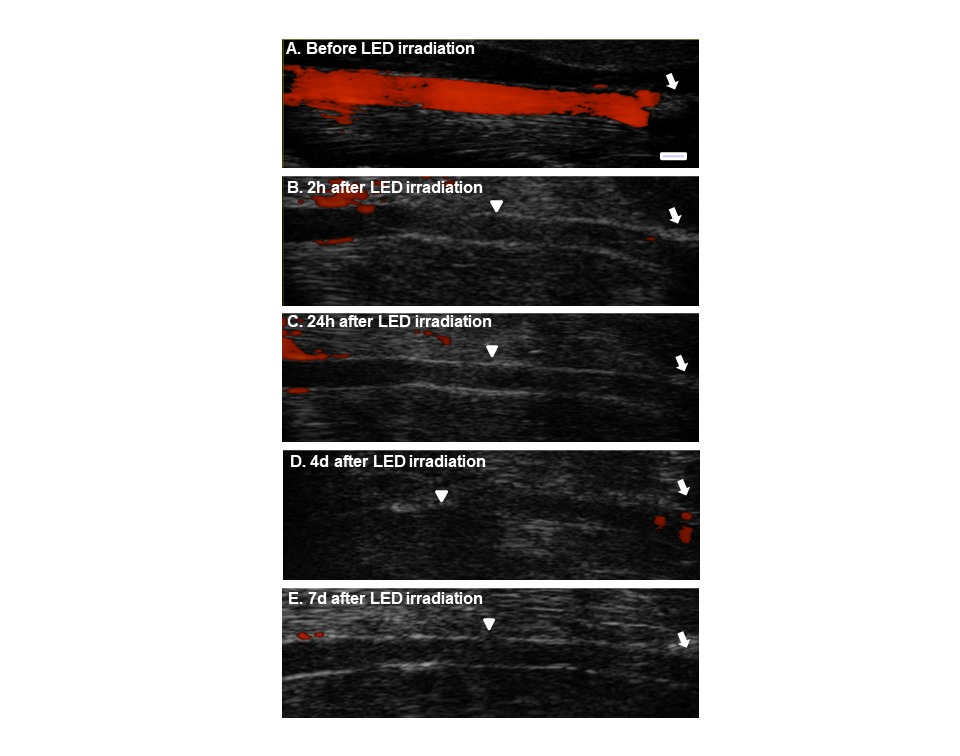

Supplement: Supplementary file 3 — Additional file 3: Fig. S3. Ultrasound studies of common carotid artery (CCA) at 5 time points before and after single LED irradiation with 6 mW/cm2 for 4 hours. (A) Before LED irradiation, there is Doppler flow (red color inside the CCA lumen). (B-E) After LED irradiation, there is no Doppler flow seen in CCA lumen and no recanalization of the occluded CCA from 2 hours, 24 hours, 4 days to 7 days. Arrow indicates the carotid bifurcation, and arrowhead indicates the stenosis segment. LED = light-emitting diode; h = hour; d = day; Scale bar in A = 1 mm. [file 13287_2021_2615_MOESM3_ESM.tif]

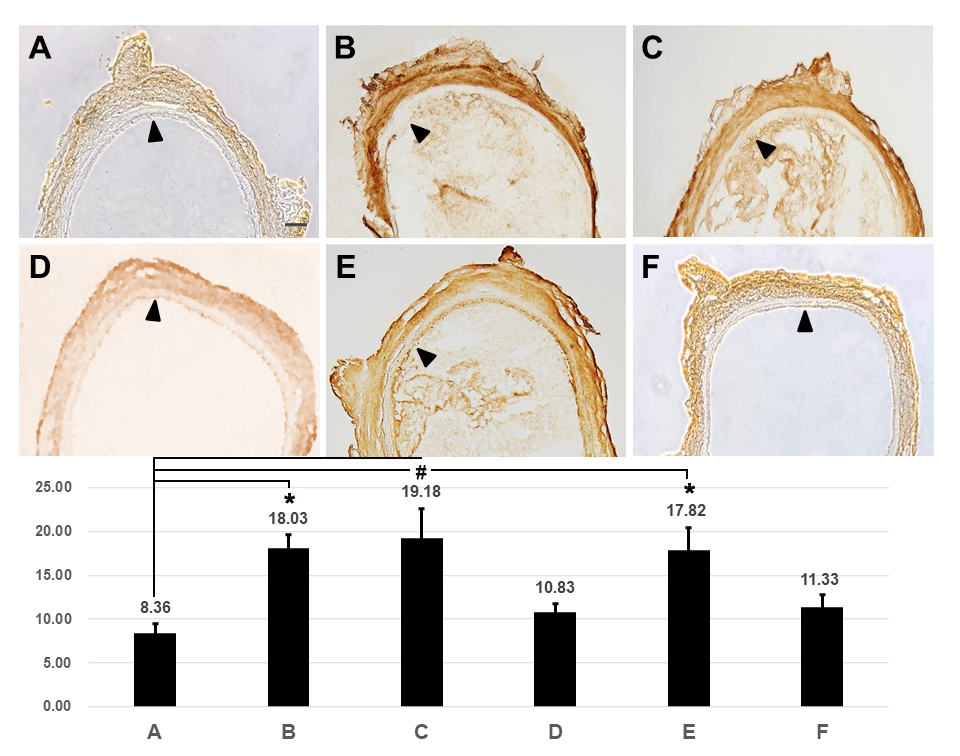

Supplement: Supplementary file 4 — Additional file 4: Fig. S4. Immunohistochemical staining of endothelin-1 (ET-1) in common carotid artery (CCA) at 7 days after single 6 mW/cm2 LED irradiation for 4 hours. Thrombus formation can be found inside CCA lumen at B, C and E. Endothelial membrane of CCA is damaged at B and C but less injured at E. However, endothelial membrane of CCA is relatively preserved at D and F. ET-1 immunoreactivity is induced significantly in LED-irradiated luminal wall of CCA at B, C and E. ET-1 immunoreactivity is less expressed in the group with iPSCs treatment (D) and is near the level of before LED irradiation (A) at F. A = before LED irradiation, B = LED irradiation alone (LED group), C = LED irradiation plus intravenous bolus injection of 0.45 mg/kg rt-PA alone (0.45 mg/kg rt-PA group), D = LED irradiation plus intravenous bolus injection of 0.9 mg/kg rt-PA alone (0.9 mg/kg rt-PA group), E = LED irradiation plus intravenous bolus injection of 1×10^6^ iPSCs alone (iPSCs group), F = LED irradiation plus intravenous bolus injection of 0.45 mg/kg rt-PA and 1 hour later, bolus injection of 1×10^6^ iPSCs (0.45 mg/kg rt-PA plus iPSCs group). LED = light-emitting diode; n = 8 in each group. Arrows indicate the endothelial membrane of CCA. Scale bar in A = 100 μm. One-way analysis of variance with Tukey–Kramer test for post-hoc comparisons is used for multiple comparison of means. *P < 0.05, #P < 0.01. [file 13287_2021_2615_MOESM4_ESM.tif]

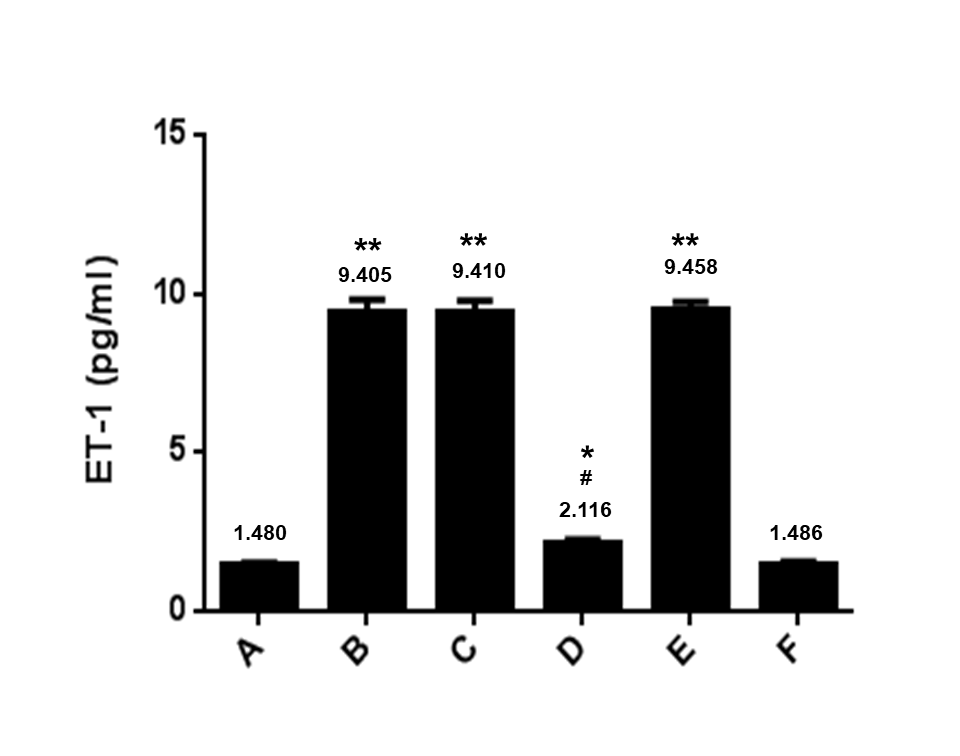

Supplement: Supplementary file 5 — Additional file 5: Fig. S5. Endothelin-1 (ET-1) level is examined using common carotid artery blood by ELISA at 7 days after single 6 mW/cm2 LED irradiation for 4 hours in each treatment group. ET-1 levels are significantly higher at B (9.405 pg/mL), C (9.410 pg/mL), and E (9.458 pg/mL) compared to A (1.480 pg/mL), D (2.116 pg/mL), and F (1.486 pg/mL) (P < 0.01). There is a significant reduction at D (2.116 pg/mL) compared to C (9.410 pg/mL) (P < 0.01). The ET-1 level at F (1.486 mg/mL) is significantly reduced, similar to that before LED irradiation (A, 1.480 pg/mL) but much lower than that at D (2.116 pg/mL) (P < 0.05). A = before LED irradiation, B = LED irradiation alone (LED group), C = LED irradiation plus intravenous bolus injection of 0.45 mg/kg rt-PA alone (0.45 mg/kg rt-PA group), D = LED irradiation plus intravenous bolus injection of 0.9 mg/kg rt-PA alone (0.9 mg/kg rt-PA group), E = LED irradiation plus intravenous bolus injection of 1×10^6^ iPSCs alone (iPSCs group), F = LED irradiation plus intravenous bolus injection of 0.45 mg/kg rt-PA and 1 hour later, bolus injection of 1×10^6^ iPSCs (0.45 mg/kg rt-PA plus iPSCs group). LED = light-emitting diode; n = 8 in each group. *P < 0.05, D vs. A group; **P < 0.01 B, C and E vs. A, D and F groups; #P < 0.05, D vs. F group. One-way analysis of variance with Tukey–Kramer test for post-hoc comparisons is used for multiple comparison of means. [file 13287_2021_2615_MOESM5_ESM.tif]
